# Two years of neurosurgical intraoperative MRI in Sweden - evaluation of use and costs

**DOI:** 10.1007/s00701-024-05978-3

**Published:** 2024-02-13

**Authors:** Magnus Kaijser, Henrik Frisk, Oscar Persson, Gustav Burström, Annika Suneson, Victor Gabriel El-Hajj, Michael Fagerlund, Erik Edström, Adrian Elmi-Terander

**Affiliations:** 1 Department of Neuroradiology, Karolinska University Hospital, Stockholm, Sweden; 2 Institute of Environmental Medicine, Karolinska Institutet, Stockholm, Sweden; 3 Department of Clinical Neuroscience, Karolinska Institutet, Stockholm, Sweden; 4 Department of Neurosurgery, Karolinska University Hospital, Stockholm, Sweden; 5 Capio Spine Center Stockholm, Löwenströmska Hospital, Stockholm, Sweden; 6 Department of Medical Sciences, Örebro University, Orebro, Sweden; 7 Department of Surgical Sciences, Uppsala University, Uppsala, Sweden

**Keywords:** MRI, Intraoperative MRI, Cost evaluation, IOMRI, Neurosurgery

## Abstract

**Background:**

The current shortage of radiology staff in healthcare provides a challenge for departments all over the world. This leads to more evaluation of how the radiology resources are used and a demand to use them in the most efficient way. Intraoperative MRI is one of many recent advancements in radiological practice. If radiology staff is performing intraoperative MRI at the operation ward, they may be impeded from performing other examinations at the radiology department, creating costs in terms of exams not being performed. Since this is a kind of cost whose importance is likely to increase, we have studied the practice of intraoperative MRI in Sweden.

**Methods:**

The study includes data from the first four hospitals in Sweden that installed MRI scanners adjacent to the operating theaters. In addition, we included data from Karolinska University Hospital in Solna where intraoperative MRI is carried out at the radiology department.

**Results:**

Scanners that were moved into the operation theater and doing no or few other scans were used 11–12% of the days. Stationary scanners adjacent to the operation room were used 35–41% of the days. For scanners situated at the radiology department doing intraoperative scans interspersed among all other scans, the proportion was 92%.

**Conclusion:**

Our study suggests that performing exams at the radiology department rather than at several locations throughout the hospital may be an efficient approach to tackle the simultaneous trends of increasing demands for imaging and increasing staff shortages at radiology departments.

## Introduction

There is an abundance of news reports of staff shortages in health care [1, 14]. For radiology, the situation is extra challenging since staff shortage is combined with an increasing demand for imaging and the training of radiologists does not keep pace with requirements [15]. In the UK alone, the Royal College of Radiologists expects the shortage of radiologists to be more than 3600 in 2025, and NHS has reported a lack of 3500 radiographers and 2000 radiologists to run its diagnostic centers [13]. In Sweden in 2022, 41% of radiographers reported having abstained from performing radiological exams because of staff shortage [10].

The increasing demand for radiology has many causes [17]. Improvements in technique have both expanded the field where radiology can contribute with accurate diagnosis and enabled the introduction of new radiology dependent treatments, such as endovascular therapy in cardiovascular and cerebrovascular disease. There is also a shift among clinicians in how imaging is used, from having been a tool to help finding the correct diagnosis to becoming a means of ruling out the highly improbable, a practice known as defensive imaging [3]. Although the relative contribution of different factors to the increasing demand for imaging may be a matter of debate, there is no questioning of the fact that, given the central role of diagnostic radiology in modern health care, any failure of radiology to meet its obligations is likely to have detrimental effects on healthcare quality. Accordingly, to allow for sound decision making on where to use the limited resource of radiologists and radiographers, there is call for the evaluation of every aspect of current radiological practices in terms of staff usage and allocation of resources.

Intraoperative MRI is one of many recent advancements in radiological practice [11]. In order to achieve more radical excision of intracranial tumors — radicality being a strong determinant of patient survival in high-grade gliomas — MRI imaging is performed during a neurosurgical procedure using dedicated scanners in or adjacent to the operating theater [7, 8, 16]. This enables the surgeon to detect and remove any remaining tumor before the operation is terminated. A recent Cochrane review [5] found that the frequency of incomplete resections was 32% without and only 4% with the use of intraoperative MRI. However, the quality of the evidence was low and based on single studies. Arguably, the rate of extended resections will be greatly influenced by the overall strategy at a given neurosurgical center: how complete resections are defined and whether the surgeons believe that improving upon near total resections will benefit the patient. While intraoperative MRI seems to increase tumor resection, its overall effect on outcomes is insufficiently studied [9]. In a systematic review and meta-analysis by Naik et al. in 2022, the authors concluded that intraoperative MRI and two methods of fluorescence guidance were all superior to non-navigated conventional surgery in achieving gross total resection. However, it is important to recognize the different roles of neurosurgical tools such as navigation, fluorescence guidance, intraoperative ultrasound, and MRI since they provide information within very different time frames [12]. These tools should be viewed as complimentary rather than competing. Available evidence suggests that the use of intraoperative MRI to increase patient survival, and in a simulation in an American setting and with an acceptable spending of 100,000 US$ per quality-adjusted life year, the method was shown to be cost-effective [12, 16]. The simulation, however, did not consider radiology staff as a limited resource. If radiology staff is employed in performing intraoperative MRI at the operation ward, they may be impeded from performing other radiological examinations at the radiology department, thus creating costs in terms of exams not being performed. Since this is a kind of cost whose importance is likely to increase, we have studied the practice of intraoperative MRI in Sweden.

## Methods

### Setting

The study includes data both from the first four hospitals in Sweden that installed MRI scanners in or directly adjacent to the operating theaters (Karolinska University Hospital Huddinge in Stockholm, Sahlgrenska University Hospital in Göteborg, and the university hospitals of Uppsala and Linköping). In addition, we included data from Karolinska University Hospital in Solna where intraoperative MRI is carried out on the MRI scanners at the radiology department. All MRI scanners in or adjacent to the operating theaters are Siemens Skyra 3 T scanners, two of which are stationary (Karolinska Huddinge and Linköping) and two have the IMRIS system, a ceiling mounted rail that enables moving the scanner into the operation theater (Sahlgrenska Göteborg and Uppsala).

The study period was from the initiation of the intraoperative scanners at each site, or for Karolinska Solna, from the start of performing intraoperative exams on regular MRI scanners to spring 2022, end of follow-up being the date when data at each university hospital was assessed. Centers, MRI scanners, and time of follow-up are presented in Table 1.

**Table 1:** MRI equipment, start, end, and days of follow-up by center

	Scanner	Start of follow-up	End of follow-up	Days of follow-up
Uppsala	Siemens Skyra Imris, 3 T	October 26, 2020	August 31, 2022	781
Göteborg	Siemens Skyra Imris, 3 T	January 1, 2021	June 2, 2022	517
Linköping	Siemens Skyra. Stationary, 3 T	March 10, 2020	April 30, 2022	781
Karolinska Huddinge	Siemens Skyra, Stationary, 3 T	February 5, 2020	May 1, 2022	816
Karolinska Solna	GE Signa 1.5 T and 3 T*	March 17, 2020	May 1, 2022	775

^*^Scanner on radiology department, not connected to operation unit

### Economic and personnel assumptions and statistics

Since the price a hospital pays for each MRI scanner normally is set by a combination of factors such as the value of old scanners being repurchased by the vendor, the number of scanners bought simultaneously, and installation costs, we used a generic price of 20 million SEK for each scanner and a 10-year depreciation period. Since service of scanners depends on time rather than usage, we assumed service costs to be equal for the scanners, 900,000 SEK/year. When calculating depreciation and service costs per exam, we multiplied these costs with study period in years and divided with number of exams performed during the study period. When calculating personnel costs, we assumed that when radiology nurses — the professional category in Sweden responsible for both caring of the patients when at the radiology department and for performing the actual scans — were scheduled whole days at the intraoperative scanner but could perform some other unscheduled tasks during this time, average time at the MRI scanner was 6 h. For salaries, we assumed the salaries for radiology nurses and assistant nurses to be the average salaries per category in 2021, 321 SEK/h and 179 SEK/h, respectively.

Since this is a descriptive study with no hypothesis testing, we abstained from statistical testing and restricted our computations to averages and sums of number of days of follow-up of each scanner, number, and proportion of days scanner used since installation. Cost per exam was calculated by dividing depreciation and service costs for the follow-up period by the number of exams performed during this period and adding staff costs according to time spent per exam per center. Since we had no information on the actual time used for the radiologists reading the scans, we did not include costs for radiologists reading the scans in our calculations.

## Results

There was a wide variation in how much the MRI scanners were used. Scanners that were moved into the operation theater and doing no or few other scans were used 11–12% of the days, whereas stationary scanners adjacent to the operation room were used 35–41% of the days. For the scanners situated at the radiology department doing intraoperative scans interspersed among all other scans, the proportion was 92%. This pattern of usage was reflected in output, which varied between 62 scans performed on one scanner in the operating theatre and 7665 scans performed on the two scanners used for intraoperative scans at the radiology department. One scanner performed no intraoperative scans at all. This was because of a reorganization of health care in the Stockholm Region during construction of the radiology department. The scanner was planned to serve spinal surgery operations, but these operations were then allocated to another hospital. Because of this, we excluded this hospital from further calculations on cost per intraoperative exam.

For radiology nurses, time spent per intraoperative exam varied more than threefold, from 2.8 to 9 h per exam. Number of exams, days scanner used, and hours allocated per intraoperative exam are presented in detail in Table 2.

**Table 2:** Number and proportion of days scanner used during follow-up, number of exams, staff allocation for intraoperative MRI exams, by center.

	Number of days scanner used	Proportion of days scanner used	Number and type of exams		Staff during intraoperative scans	Hours allocated per intraoperative exam	
						*Radiology nurse*	*Assistant nurse*
Uppsala	77	11%	77	- 77 intraoperative	1 extra assistant nurse in operation team 2 radiology nurses whole day with interceptions for intraoperative cancer surgery etc., approx. 2 h per radiology nurse for DBS	8.4	5.3
Göteborg	62	12%	62	- 47 intraoperative - 15 outpatient research	1–2 radiology nurses whole day with interceptions for intraoperative patients, 1 radiology nurse 1 h for research patients	9	-
Linköping	276	35%	760	- 60 Intraoperative - 571 neurosurgical and neurointensive inpatients - 129 outpatient research	1 assistant nurse and 1 radiology nurse whole day with interceptions for intraoperative exams	6	6
Karolinska Huddinge	331	41%	1355	- 0 intraoperative - 620 in general anesthesia - 412 other clinical exams - 323 outpatient research exams	Not applicable	Not applicable	Not applicable
Karolinska Solna	1429**	92%	7665	- 64 intraoperative, of which - 34 LITT - 30 other intraoperative - 7601 in- and outpatient exams	1 radiology nurse 4 h for LITT, 1–2 radiology nurses 1 h for other intraoperative scans	2.8	-

^**^Two scanners

In the calculation of costs, scanner costs per intraoperative exam varied between 1110 SEK per exam to 47,960 SEK per exam, and there was a 29-fold variation in total costs for the intraoperative scans, when excluding radiologist reading time, between 2520 SEK and 73,180 SEK per exam. Costs per intraoperative exam are presented in further detail in Table 3.

**Table 3:** Costs per intraoperative MRI exam by center

	Cost MRI scanner/exam	MRI service/exam	Staff costs*	Total cost/exam
	*20 million Skr/scanner,* *10-year depreciation period*	*900,000 Skr/year*	*Radiology nurse 321 Skr/h* *Assistant nurse 179 Skr /h*	Scanner and staff costs*
Uppsala	47,960	21,580	3630	73,180
Göteborg	45,690	20,560	2890	69,150
Linköping	5630	2530	3000	11,160
Karolinska Solna	1110	500	900	2520

^*^Radiologist reading not included

## Discussion

In this study, we found that compared to intraoperative exams on scanners at the radiology department, performing intraoperative MRI exams on scanners built for exclusive use in the operation theater was up to 29 times more expensive. Another finding of the study was a more than threefold reduction in time needed for radiology nurses per exam when performing the exams at the radiology department compared to at the operation ward.

Our study has many shortcomings. First and foremost is the lack of detailed data on personnel time spent per exam. Instead, we have relied on assumptions based on scheduling practices on each radiology department. It is possible that with individual personnel data on each exam the results would have been modified, but we find it highly unlikely that the huge differences in costs found in our study could be explained by lack of precision in the data on personnel allocation. Another shortcoming is that we have assumed the costs for MRI scanners and service to be equal among the departments, which is certainly not the case. We argue, however, that including peripheral costs such as status of scanners repurchased by the scanner vendor or multicamera installation discounts into our calculations would have introduced more errors into the calculations than assuming scanner costs being the same. A third shortcoming is that we have some differences in follow-up periods for the different sites. The reasons for this were practical; data collection was performed on each site when feasible for the authors. We consider it, however, to be inconceivable that these differences in the length of follow-up could have any important impact on the overall integrity of the data.

Intraoperative MRI has been shown to be a valuable tool in the treatment of intracranial tumors, and its role in modern care is likely to increase in the future. Such a development, however, depends on costs being justifiable. Even though studies have found that intraoperative MRI may be cost effective [2], this may change with the increasing scarcity of radiology personnel. Our findings point to costs hidden in the suboptimal use of scanners and personnel. with low usage of the scanners in combination with ineffective usage of the radiology staff which cannot run other scanners while deployed in the OR. It is promising, though, to find that costs for intraoperative exams can be substantially reduced when performed within the regular workflow of the radiology department.

Several studies have found that placing radiology equipment nearer to the patients, such as placing CT scanners at ER departments, may increase both patient turnover and safety [6]. Importantly, even though intraoperative MRI prolongs surgery and imposes the need to temporarily close the surgical wound, available studies have found no increase in the rate of surgical infections [4, 18]. However, there are no studies on intraoperative MRI outside the neurosurgical department. Although CT scanners and ER departments are outside the scope of our study, our results may suggest that performing exams at the radiology department is more cost effective, and, accordingly, that breaking up the integrity of a radiology department and placing scanners on multiple locations throughout a hospital may have negative effects on imaging capacity overall. In addition, prioritizing the integrity of the radiology department may serve to prevent costly investments in technology soon to be abandoned. Arguably, the upkeep and maintenance of radiological equipment are best performed at a radiological department. Moreover, resources spent at the radiological department may safeguard healthcare providers from mistaken investments as priorities of the organization change.

In conclusion, we have found that the costs of performing intraoperative MRI in the operation room compared to at the radiology department may be up to 29 times higher and may demand more than three times as much time from radiology nurses per exam. Our study suggests that performing exams at the radiology department rather than at several locations throughout the hospital may be an efficient approach to tackle the simultaneous trends of increasing demands for imaging and increasing staff shortages at radiology departments.

## Data Availability

Data is available upon request from the corresponding author.

## Code availability

Code availability is not applicable.
